# Obnet: Network of semantic associations for obesity

**DOI:** 10.1186/1471-2105-15-S10-P6

**Published:** 2014-09-29

**Authors:** Hossein Taghizad, Mohammad Yeasin, Tara Cherry, Vida Abedi

**Affiliations:** 1 College of Arts and Sciences, Bioinformatics program, University of Memphis, Memphis, TN 38152, USA; 2 Depatrment of Electrical and Computer Engineering, University of Memphis, Memphis, TN 38152, USA; 3 Department of Graduate Health Science, University of Tennessee Health Sciences Center, Memphis, TN 38163, USA

## Background

Obesity is a complex disease that causes health complications and mortality. Recently, the Centers for Disease Control (CDC) classified obesity as a disease and introduced guidelines for diagnosis. To understand complex relationships among all contributing factors, we have developed the ObNet (Network of Semantic Association for Obesity). The concepts were adopted from ARIANA [1,2]. The Local Latent Semantic Analysis tool [3], developed in the CVPIA lab, was used to extract network of semantic associations. Web services and visualization were developed to display the associations in a flexible network form that is easy to interact.

## Materials and methods

In this pilot study, 198 Medical Subject Headings (MeSH) terms were selected as potential risk factors by a clinician that specializes in weight gain and obesity. Abstracts from the PubMed database were mined to create the semantic model. A Query-Based Sampling (QBS) process [3] was used to create balanced and “Representative Samples” for modeling the ObNet. A relevance model [1] was adopted to categorize associations in three groups: Clear Association, Established Concepts, and Current Research Topics.

## Results

ObNet is a web service that provides users with the network of semantic associations for obesity, using literature data. The system can be queried with an array of keywords related to obesity to extract a customized network of associated entities. A pilot study using Obnet revealed current research topics in obesity. Three terms were identified and evaluated by a field expert: dietary fat, adults, and attitude to health. Using a direct literature search, corroborating evidence was found to indicate the three identified terms were current research topics. First, Tan et al. [4] investigated the anti-metabolic disorder effects of kumquat (*Fortunella margarita* Swingle) fruit extract (FME) on high-fat diet-induced C57BL/6 obese mice. Second, in another study, 149 primary care physicians were surveyed and their knowledge and perceived barriers in the management of overweight and obesity were measured [5]. Finally, in a recent investigation, the impacts of neighborhood park access and quality on body mass index among adults in New York City were measured [6].

## Conclusions

The ObNet is an innovative web service tool for the assessment of obesity. It has the potential to support diagnosis and uncover novel relationships with the disease. Empirical study suggests that the model is capable in finding network of concepts and current research trends for obesity related keywords. Future direction of the study will improve the visualization module, and develop a mobile application.
